# In Silico Analysis and Expression Profiling of Adhesin-Like Flocculins in *Trichosporon asahii*

**DOI:** 10.1007/s11046-025-01045-2

**Published:** 2026-02-02

**Authors:** Mateus Jorge Mendes dos Santos, Danielle de Castro Quinto, Guilherme de Souza Gagliano, Elaine Cristina Francisco, Ana Carolina Barbosa Padovan

**Affiliations:** 1 Graduate Program in Fungal Biology, Federal University of Pernambuco, Recife, Brazil; 2 Department of Microbiology and Immunology, Institute of Biomedical Sciences, Universidade Federal de Alfenas/UNIFAL-MG/Room Q113G, Gabriel Monteiro da Silva, 700 Alfenas, Minas Gerais, 37130-001 Brazil; 3 Special Mycology Laboratory, Division of Infectious Diseases, Paulista School of Medicine, Federal University of São Paulo, São Paulo, Brazil; 4 Center for Monitoring Antimicrobial Resistance Patterns and Genetic Diversity in Clinical and Environmental Isolates from Brazilian Biomes, Universidade Federal do Paraná, Curitiba, Brazil; 5 Antimicrobial Resistance Institute of São Paulo (ARIES), São Paulo, Brazil

**Keywords:** Infection, Virulence factor, Adhesin, Human proteins, *Trichosporon asahii*

## Abstract

**Background:**

*Trichosporon asahii* is an opportunistic basidiomycetous yeast increasingly recognized as a cause of invasive infections in immunocompromised individuals, often associated with high morbidity and mortality. Its ability to adhere to host tissues and abiotic surfaces is considered a critical virulence factor, yet the molecular mechanisms underlying this adhesion remain poorly understood.

**Objective:**

To characterize gene sequences in *T. asahii* belonging to the flocculin family with potential adhesive functions.

**Methods:**

The homologous flocculin genes *DHA1, DHA2, CPL1,* and *CFL1*, previously described in *Cryptococcus neoformans* as being involved in cell adhesion, were identified within the *T. asahii* predicted proteome. Four *T. asahii* strains were analyzed: two reference strains (CBS2479 and CBS7631) and two clinical strains (L2585 and L773). Cells were grown in planktonic morphology and biofilms in RPMI + MOPS medium with 2% glucose. The predominant morphology of the cells was observed using bright-field optical microscopy, and total RNA was extracted. The relative expression of the four flocculin genes was analyzed by qRT-PCR and normalized using the 2-ΔΔCt method and the actin gene.

**Results:**

The flocculin proteins showed high similarity and common domains with *C. neoformans* flocculins and other adhesins. The strains exhibited a variety of morphologies, with the *T. asahii* CBS2479 strain predominantly displaying yeast forms, while the *T. asahii* L2585 strain presented a higher quantity of hyphae. All flocculins bound to human proteins in the PriaA/Cpl1_fungi domain and/or to the Cpl1-like domain. *T. asahii* flocculins were downregulated when compared to the reference strain CBS2479, while the clinical strain L2585 exhibited the highest expression of the flocculin *CFL1*. During biofilm formation, *DHA1* and *CFL1* were highly expressed.

**Conclusion:**

Flocculins were expressed according to specific yeast/hyphal morphology and were also more expressed in biofilms, indicating their association with biofilm maintenance and development.

**Supplementary Information:**

The online version contains supplementary material available at 10.1007/s11046-025-01045-2.

## Introduction

*Trichosporon* spp*.* are yeasts-like basidiomycetes that, while commonly part of the human microbiota, can act as opportunistic pathogens, causing life-threatening infections particularly in immunocompromised hosts. Among the species within this genus, *Trichosporon asahii* is the most frequently identified in clinical settings, accounting for approximately 73–85.7% of *Trichosporon-*related infections [27, 29, 35]. Invasive trichosporonosis is associated with high mortality rates, ranging from 42 to 92% in immunocompromised and neutropenic hosts. Despite this relevance, little is known about the virulence mechanisms of this species [4, 14, 16, 35].

The pathogenicity of *T. asahii* is multifactorial, involving a range of virulence traits that enhance its survival and persistence in the host. These include secretion of hydrolytic enzymes such as proteases, lipases, DNAses, and phospholipases, which facilitate tissue invasion and immune evasion [33]. In addition, the production of melanin and expression of heat shock proteins contribute to the organism's resistance against host defenses and environmental stressors [2, 13, 32, 33]. Furthermore, *T. asahii* demonstrates the ability to form robust biofilms, posing an important challenge to its eradication due to increased resistance to antifungal agents and immune clearance [5, 27].

Among the key virulence factors in fungal pathogens are adhesins, surface proteins that mediate the initial attachment to host cells and abiotic surfaces. These molecules are critical for colonization, invasion, and the establishment of biofilms [3]. A prominent subclass of adhesins are Flocculins, initially characterized in *Saccharomyces cerevisiae* for their role in cell–cell adhesion and floc formation. Homologous proteins with adhesive functions have since been identified in other pathogenic yeasts, including *Candida* spp. and *Cryptococcus* spp. [3, 21, 26, 30]. Considering that adhesins are central to fungal adherence, colonization, and biofilm formation, the identification and characterization of flocculins with putative adhesive function in *T. asahii* represent a critical gap in our current knowledge.

This study therefore aims to characterize flocculin-encoding genes in *T. asahii*, investigate their structural and potential functional properties, and evaluate their expression under biofilm-inducing conditions, offering new insights into the adhesion mechanisms of this emerging fungal pathogen.

## Material and Methods

### Strains and Microscopy Assays

Four *Trichosporon asahii* isolates were analyzed: two reference strains, CBS2479 (psoriasis lesion) and CBS7631 (blood), and two clinical strains, L773 and L2585 (blood cultures from different patients). The strains were stored at -80ºC in Yeast Extract Peptone Dextrose (YEPD) medium supplemented with 20% glycerol. Prior to experiments, the strains were reactivated on Sabouraud Dextrose Agar (SDA), and purity was confirmed using CHROMagar® Candida (Chromagar, Difco, France) after 48 h of incubation at 35 ± 2 °C. For morphological examination, cells grown under planktonic conditions were mounted on glass slides and visualized by bright-field microscopy (Q Color 5, Olympus America) at 400 × magnification.

### Identification of *Trichosporon asahii* Adhesin-like Proteins

Four *Cryptococcus neoformans* proteins with known or putative adhesin function, Dha1p, Dha2p, Cpl1p, and Cfl1p, were selected based on prior literature [21, 41]. The corresponding NCBI identifiers were CNAG_07422, CNAG_06082, CNAG_02797, and CNAG_00795, respectively. Homology searches were conducted against the translated *T. asahii* genome using t-BLASTn (https://blast.ncbi.nlm.nih.gov/Blast.cgi). Sequences with ≥ 50% identity and/or coverage were considered putative homologs.

### Phylogenetic Analysis

All flocculin DNA sequences from *C. neoformans* and *T. asahii* that encoded the respective protein sequences were downloaded from NCBI-GenBank. After intron removal, the sequences were aligned using the Muscle algorithm implemented by the SeaView 5.0.5 program [17]. A Maximum Likelihood phylogenetic tree was inferred using the GTR + I + Γ model [18, 40, 42]. Bootstrap analysis (bt) [10] was conducted by evaluating 100 pseudoreplicates of the alignment. All analyses were computed in the SeaView 5.0.5 program [17].

### In silico Characterization of *T. asahii* Flocculins

Predicted flocculin sequences in *T. asahii* were analyzed for features associated with adhesion: signal peptide, GPI-anchor, mannosylation, *N*- and *O*-linked glycosylation, and acetylation. These analyses were performed using prediction tools from the Center for Biological Sequence Analysis (CBS) at DTU Health Tech (http://www.cbs.dtu.dk/services/). Protein families and conserved domains were identified using InterPro (https://www.ebi.ac.uk/interpro/).

Three-dimensional protein structure prediction was performed using AlphaFold (https://310.ai/docs/docking). PDB files corresponding to the predicted flocculins Dha1p (J6EUW3), Dha2p (J4UDD2), Cpl1p (J4U5C9), and Cfl1p (J4U8G0)—and human extracellular matrix proteins (albumin [1AO6], collagen [1LI1], fibronectin [1E88], hemoglobin [1A3N], and laminin [5XAU]) were retrieved from the Protein Data Bank. Molecular docking was conducted using ClusPro 2.0 (https://cluspro.org/home.php). The best binding conformations were selected based on the platform’s energy-based scoring formula: E = 0.40xErep +  − 0.40xEatt + 600xEelec + 1.00xEDARS.

### Induction of Planktonic and Biofilm Morphologies

Colonies from cultures on Sabouraud Dextrose Agar (SDA) were inoculated in 20 mL of liquid RPMI + MOPS medium with 2% glucose and incubated overnight, at 37 °C with agitation at 180 rpm. Cells were then pelleted and washed with cold PBS to proceed to RNA extraction.

Biofilm formation was induced based on a modified protocol from Iturrieta-Gonzalez [24]. Briefly, cells were cultivated overnight in mL of RPMI + MOPS medium under the same conditions described above. After harvesting and PBS washing, suspensions of 10^7^ cells/mL were prepared and inoculated into 75 cm^2^ culture flasks containing 15 mL of fresh RPMI-MOPS with 2% glucose. Adhesion was allowed for 90 min at 37 °C and 75 rpm. Non-adherent cells were removed via PBS washes, and fresh medium was added. Biofilms were developed over 22 h at 37 °C with gentle agitation (30 rpm). After incubation, media were aspirated, and cells were washed with PBS. Biofilm-forming cells were harvested using sterile scrapers for downstream RNA extraction.

### RNA Extraction and cDNA Synthesis

Total RNA was extracted using Trizol® reagent (Invitrogen, Carlsbad, CA, EUA) with mechanical disruption via glass beads (425–600 µm) and TissueLyser (Qiagen) at 45 Hz for 8 cycles of 1 min, interspersed with 1 min on ice. The RNA’s quality was confirmed by agarose gel electrophoresis (1.5%), and concentrations were determined using a DeNovix spectrophotometer (DeNovix, Wilmington, DE, USA Samples was treated with DNase I (RQ1 RNase-Free DNase, Promega, Madison, WI, USA) and cDNA was synthesized using MMLV reverse transcriptase (Promega, Madison, WI, USA) following the manufacturer’s instructions.

### qPCR Assay

Gene expression was performed using the SYBR® Select Master Mix kit (Promega, Madison, WI, USA) on a StepOnePlus Real-Time PCR System (Applied Biosystems, Foster City, CA, USA). Primers for adhesin-encoding genes and the reference gene (actin) are listed in Supplementary Table 1. Relative quantification was calculated using the 2^−ΔΔCt^ method. Statistical analyses were performed using the one-way ANOVA followed by Bonferroni post-hoc correction.

## Results

### Comparative Sequence Analysis Reveals Conserved Cpl1-like Domains Among *Trichosporon asahii *and *Cryptococcus neoformans*

To explore potential functional homology between *Trichosporon asahii* and *Cryptococcus neoformans* flocculins, we performed pairwise comparisons of four surface-associated proteins: Dha1p, Dha2p, Cpl1p, and Cfl1p. Sequence identity and coverage percentages are summarized in Table 1.

**Table 1:** Percentages of identity and coverage of *Cryptococcus neoformans* H99 *versus Trichosporon asahii* CBS2479 flocculins, respectively

		*Cryptococcus neoformans*			
		Dha1p	Dha2p	Cpl1p	Cfl1p
*Trichosporon asahii*	Dha1p-like	53% and 33,71%	53% and 33,52%	-	27% and 37,50%
	Dha2p-like	44% and 33,33%	81% and 30,26%	-	55% and 26,23%
	Cpl1p-like	31% and 29,52%	67% and 58,24%	87% and 22,11%	57% and 31,30%
	Cfl1p-like	31% and 23,97%	31% and 26,45%	34% and 30,67%	68% and 59,12%

-: indicates absence. Dha1 (Delayed-type hypersensitivity antigen-related protein) and Cpl1p (Pria protein)

The highest identity (87%) was observed between *T. asahii* Cpl1p and its *C. neoformans* ortholog, despite a lower alignment coverage (22.11%). Dha2p also exhibited strong conservation (81% identity over 30.26% coverage) with its respective homolog. In contrast, Dha1p exhibited the lowest overall sequence identity, with a maximum of 53% across 34% of the alignment. Notably, due to the high degree of conservation among sequences in *T. asahii*, all four homologous proteins consistently appeared in the BLAST analyses, regardless of the specific *C. neoformans* flocculin used as the query. Only Cpl1p from *C. neoformans* showed undetectable identity to the Dha1p-like and Dha2p-like proteins of *T. asahii*.

To evaluate the conservation of flocculin DNA sequences, we performed a phylogenetic analysis. The Maximum Likelihood tree, with a score of ln(L) = –8393.51396, revealed that the *DHA1* and *DHA2* genes are paralogous between *C. neoformans* and *T. asahii*, whereas *CFL1* and *CPL1* are homologous orthologues, supported by bootstrap confidence values above 70 (Supplementary Fig. 1).

### Post-Translational Modification Profiles Suggest Adhesin-Like Functions

Predicted post-translational modifications (PTMs) associated with adhesion activity were assessed using DTU Health Tech servers (Table 2). All four *T. asahii* proteins presented signal peptides and were predicted to be extracellular, suggesting complete secretion or membrane anchoring. *O*-linked glycosylation was also present in all sequences tested, consistent with known features of fungal cell surface adhesins.

**Table 2:** Predicted post-translational modifications of *Trichosporon asahii* flocculins according to the tools of DTU Health Tech:

	Mannosylation	Acetylation	*N*-linked Glycosylation	*O*-linked Glycosylation	Signal Peptide	GPI Anchor
Dha1p-like	x	-	x	x	x	-
Dha2p-like	-	-	x	x	x	x
Cpl1p-like	-	-	-	x	x	-
Cfl1p-like	-	-	x	x	x	-

X: indicates presence, and - indicates absence

*N*-linked glycosylation was predicted in all proteins except Cfl1p. Mannosylation was detected solely in Dha1p, while a glycosylphosphatidylinositol (GPI) anchor was exclusive to Dha2p. None of the proteins exhibited signs of acetylation. Interestingly, Cpl1p was the only candidate predicted to harbor a transmembrane domain, indicating a potential role as a membrane ligand.

### Conserved Structural Domains Support Functional Similarity Within the PriA/Cpl1_fungi Family

**Fig. 1:**
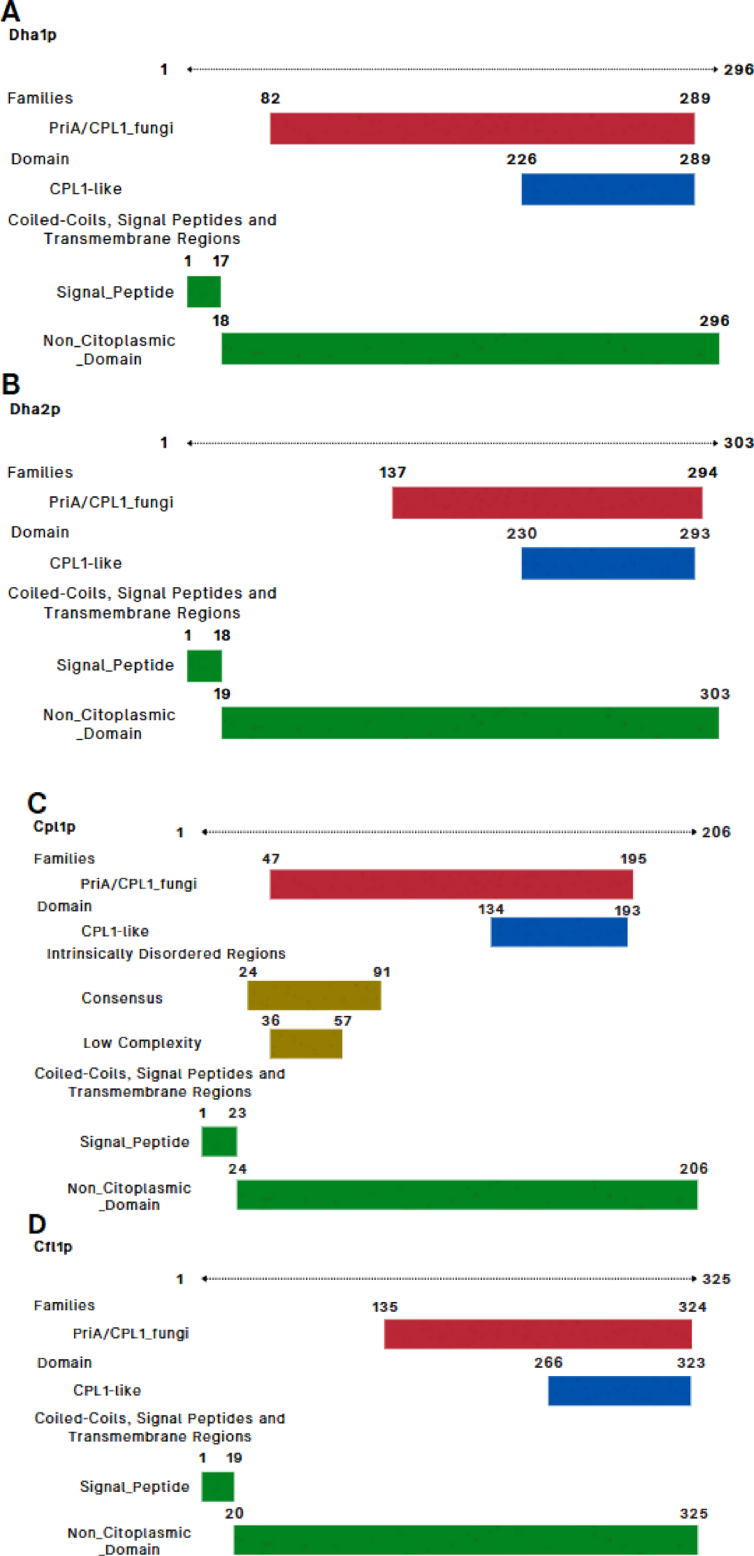
Homologous flocculins in *Trichosporon asahii* with conserved domains depicted. **A** Dha1p; **B** Dha2p; **C** Cpl1p; and **D** Cfl1p

All four *T. asahii* proteins were classified within the InterPro family IPR038955 and the corresponding PANTHER family PTHR35192, associated with the PriA/Cpl1_fungi subfamily (Fig. 1). Each protein also contained the Cpl1-like domain (IPR048661, Pfam PF21671), located in the C-terminal region (residues ranging from 47–324), consistent with domain boundaries observed in fungal adhesins (Fig. 1).

Dha1p and Dha2p shared similar domain organizations, with both proteins harboring signal peptides and extracellular regions spanning the Cpl1-like domain. Cpl1p was unique in that, in addition to the signal peptide and Cpl1-like domain, it featured intrinsically disordered regions (residues 24–91) and a low-complexity segment (residues 36–57), features often associated with flexible binding interfaces.

Cfl1p, although less conserved in terms of sequence identity, retained the Cpl1-like domain and presented a disordered region (residues 193–215), possibly contributing to its functional plasticity. According to UniProt annotations, Dha1p and Dha2p are homologous to delayed-type hypersensitivity antigens, while Cpl1p is functionally related to the Pria protein, and Cfl1p contains domains similar to Cpl1p.

### *Trichosporon asahii* Flocculins Exhibit High-Affinity Interactions with Human Extracellular and Plasma Proteins

To investigate the potential role of *T*. *asahii* flocculins in host–pathogen interactions, we performed three-dimensional (3D) structural modelling of four adhesin candidates, Dha1p (J6EUW3), Dha2p (J4UDD2), Cpl1p (J4U5C9), and Cfl1p (J4U8G0), followed by molecular docking analyses with five representative human proteins: albumin, hemoglobin, fibronectin, collagen IV, and laminin.

All flocculin structures were predicted within cluster 0 (Supplementary Table 2). Among them, Cfl1p showed the best structural modelling scores, with a central score of –1224.9 and a minimum energy of –1254.0, suggesting a high degree of conformational stability. Structurally, all four proteins exhibited typical secondary elements such as alpha-helices, beta-sheets, and coils. Notably, the conserved PriA/Cpl1_fungi motifs in each flocculin contained a combination of beta-sheets and alpha-helices, indicating that these structural features are compatible with proper protein folding and domain integrity.

Subsequent molecular docking analyses revealed that all flocculins were capable of forming stable complexes with the tested human targets (Supplementary Tables 2–6). The highest minimum binding was found to Cfl1p interacting with all human proteins (–1605.4 in cluster 0), whereas Dha1p exhibited values up to –894.7 (cluster 3) when interacting with fibronectin. These results suggest a variable but consistently high-affinity binding potential across all flocculins.

To better understand the mechanisms by which *T. asahii* flocculins recognize and bind to host targets, we mapped the residue-level interaction interfaces between each adhesin and five representative human proteins: albumin, collagen IV, fibronectin, hemoglobin, and laminin. Particular attention was given to identifying the specific structural domains involved in these interactions, especially to the conserved PriA/Cpl1_fungi and Cpl1-like motifs. The binding patterns revealed a domain-dependent interaction profile, with Dha1p and Cpl1p frequently engaging the PriA/Cpl1_fungi region, while Dha2p and Cfl1p predominantly utilized N-terminal or Cpl1-like domains. These results demonstrate that flocculin-host interactions are structurally organized rather than random, with conserved residues contributing to target specificity across different host proteins.

**Fig. 2:**
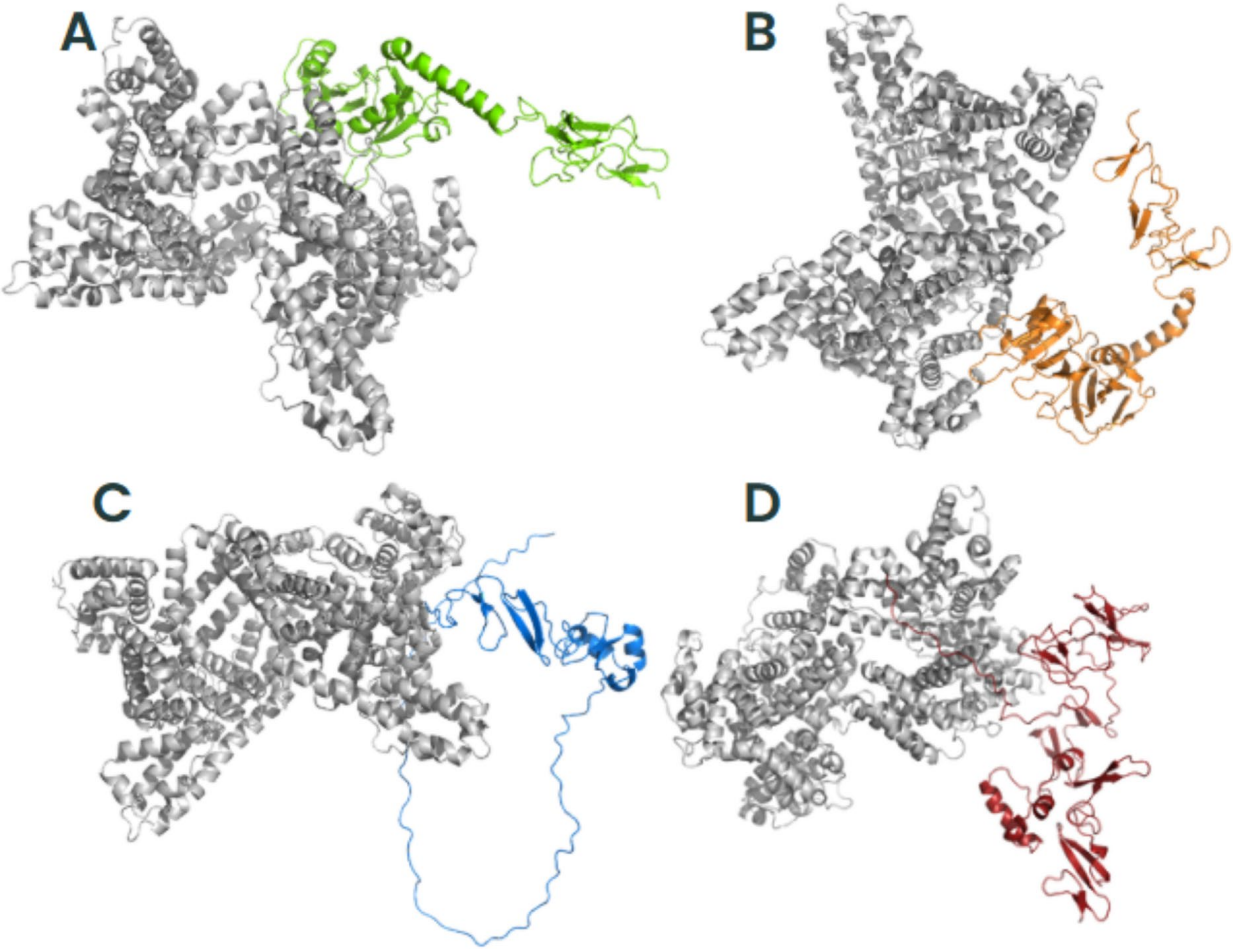
Flocculins binding to the human protein albumin in gray. **A** Dha1p in green; **B** Dha2p in orange; **C** Cpl1p in blue; and **D** Cfl1p in red

**Fig. 3:**
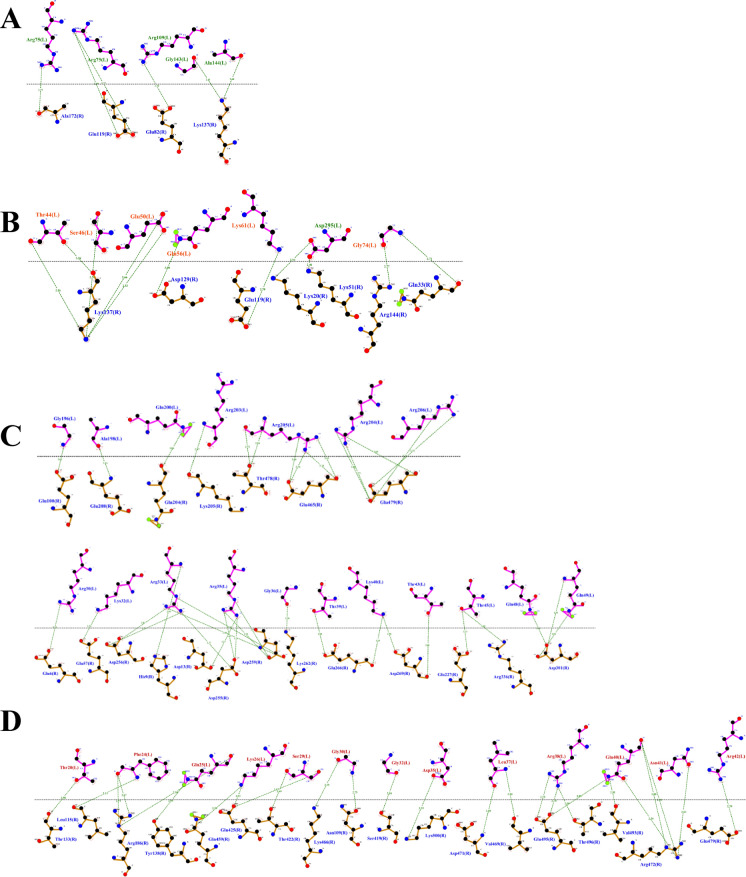
Binding between amino acid residues of human albumin, in blue, and those of the fungal flocculins A Dha1p in green; **B** Dha2p in orange; **C** Cpl1p in blue; and **D** Cfl1p in red

The interaction interfaces between *T. asahii* flocculins and human albumin (Figs. 2 and 3) were primarily located within the PriA/Cpl1_fungi domain of Dha1p (Arg75, Arg109, Gly143, Ala144) and Cpl1p (Gln48, Gln49). In contrast, albumin binding to Dha2p and Cfl1p was mediated by residues located in the N-terminal regions, including Thr44, Ser46, Glu50, Gln56, Lys61, and Gly74 for Dha2p, and Thr20, Phe24, Gln25, Lys26, Ser29, Gly30, Gly32, Asp35, Leu37, Arg38, Gln40, Asn41, and Arg42 for Cfl1p.

**Fig. 4:**
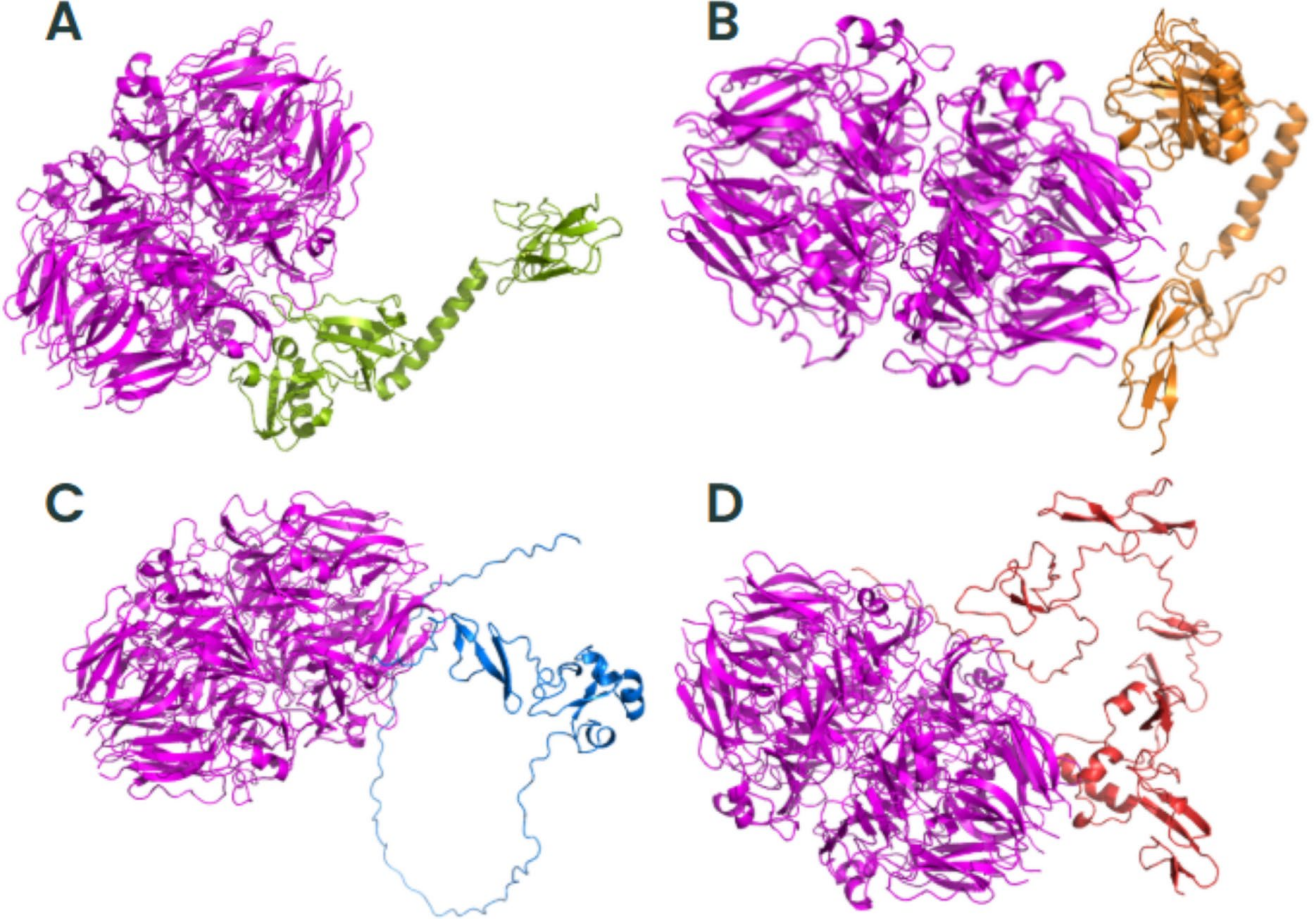
Flocculins binding to the human collagen IV protein in pink. **A** Dha1p in green; **B** Dha2p in orange; **C** Cpl1p in blue; and **D** Cfl1p in red

**Fig. 5:**
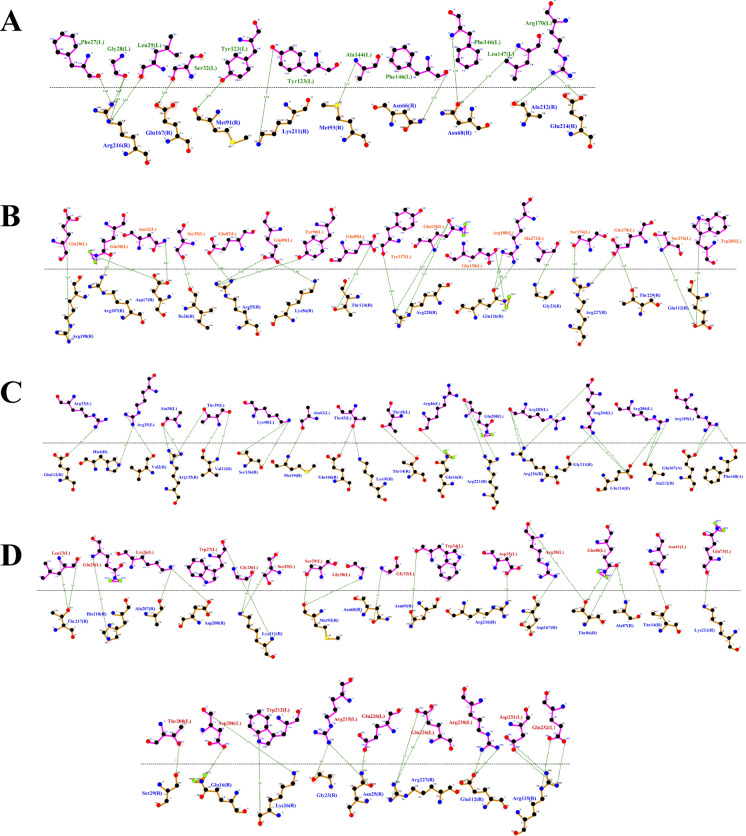
Binding between amino acid residues of human collagen IV in blue, and those of the fungal flocculins. **A** Dha1p in green; **B** Dha2p in orange; **C** Cpl1p in blue; and **D** Cfl1p in red

For collagen IV (Figs. 4 and 5), Dha1p interacted through its PriA/Cpl1_fungi domain (Thr123, Ala144, Phe146, Leu147, Arg170), while Cfl1p bound via C-terminal residues (Asp206, Thr208, Trp212, Arg215, Glu226, Arg230, Asp231, Glu232). Dha2p showed interactions in both the N-terminal portion of the conserved domain (Tyr137, Gln138, Glu158, Arg180) and the Cpl1-like domain (Ala272, Ser276, Glu278, Trp285). Cpl1p exhibited interaction residues dispersed across both the N-terminal and C-terminal regions.

**Fig. 6:**
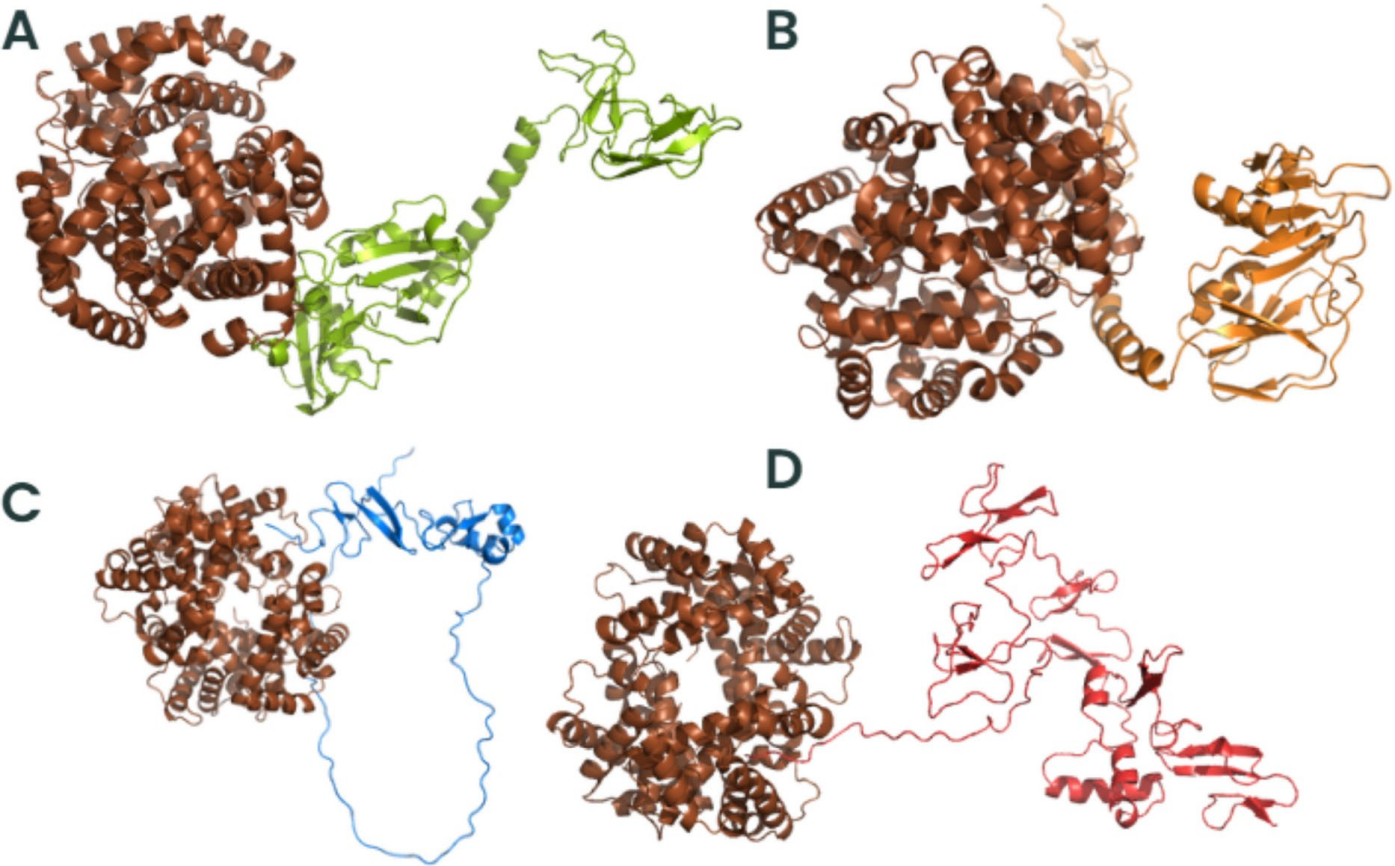
Flocculins binding to the human protein fibronectin in yellow. **A** Dha1p in green; **B** Dha2p in orange; **C** Cpl1p in blue; and **D** Cfl1p in red

**Fig. 7:**
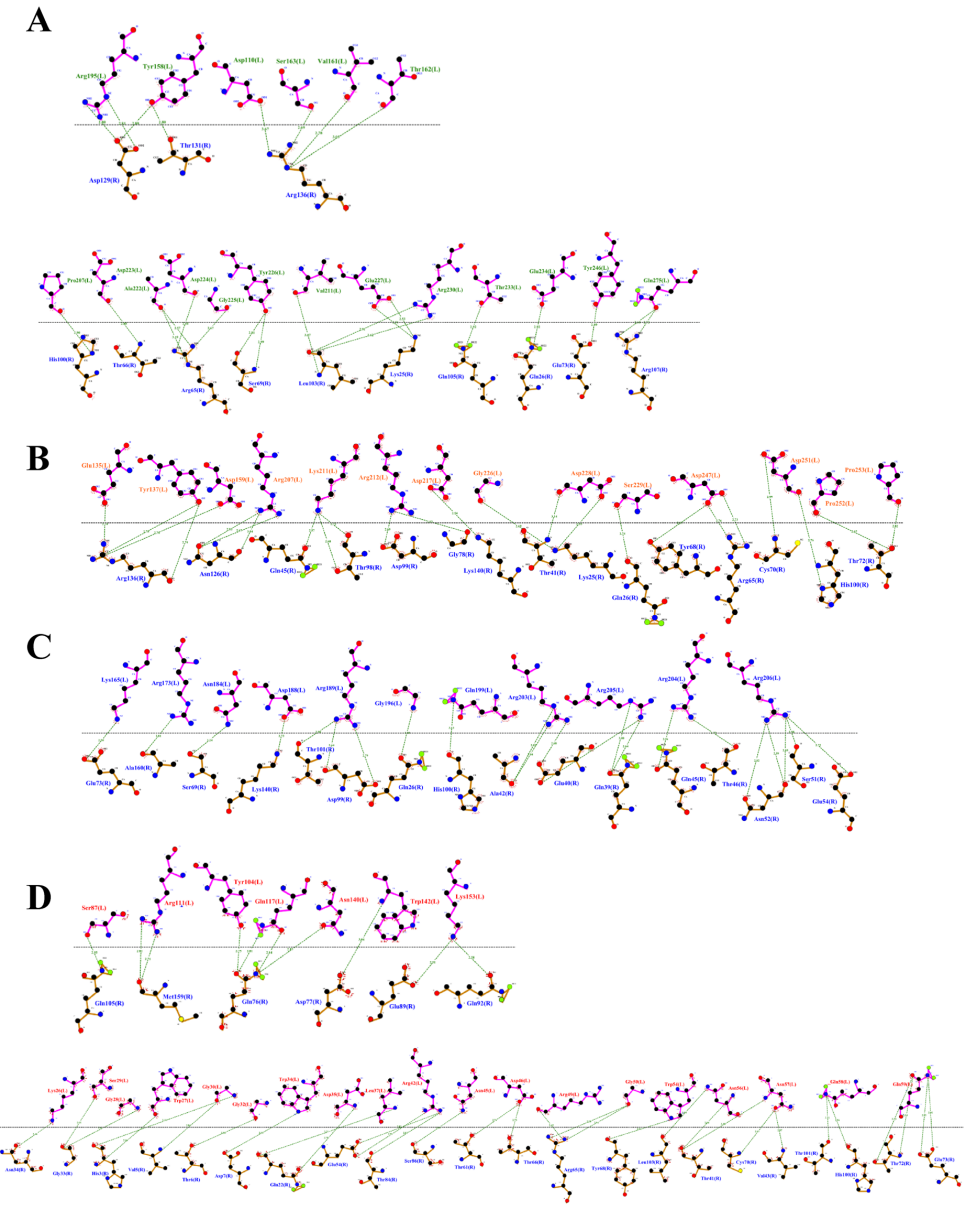
Binding between amino acid residues of human fibronectin in blue, and those of the fungal flocculins. **A** Dha1p in green; **B** Dha2p in orange; **C** Cpl1p in blue; and **D** Cfl1p in red

In the case of fibronectin (Figs. 6 and 7), Dha1p interacted with residues spanning both the PriA/Cpl1_fungi and Cpl1-like domains (Thr226, Glu227, Arg230, Thr233, Glu234, Thr246, Gln275). Cfl1p also displayed binding in the N-terminal region and the conserved domain (Asn140, Trp142, Lys153). Dha2p showed predominant binding through the Cpl1-like domain, while Cpl1p engaged both terminal regions.

**Fig. 8:**
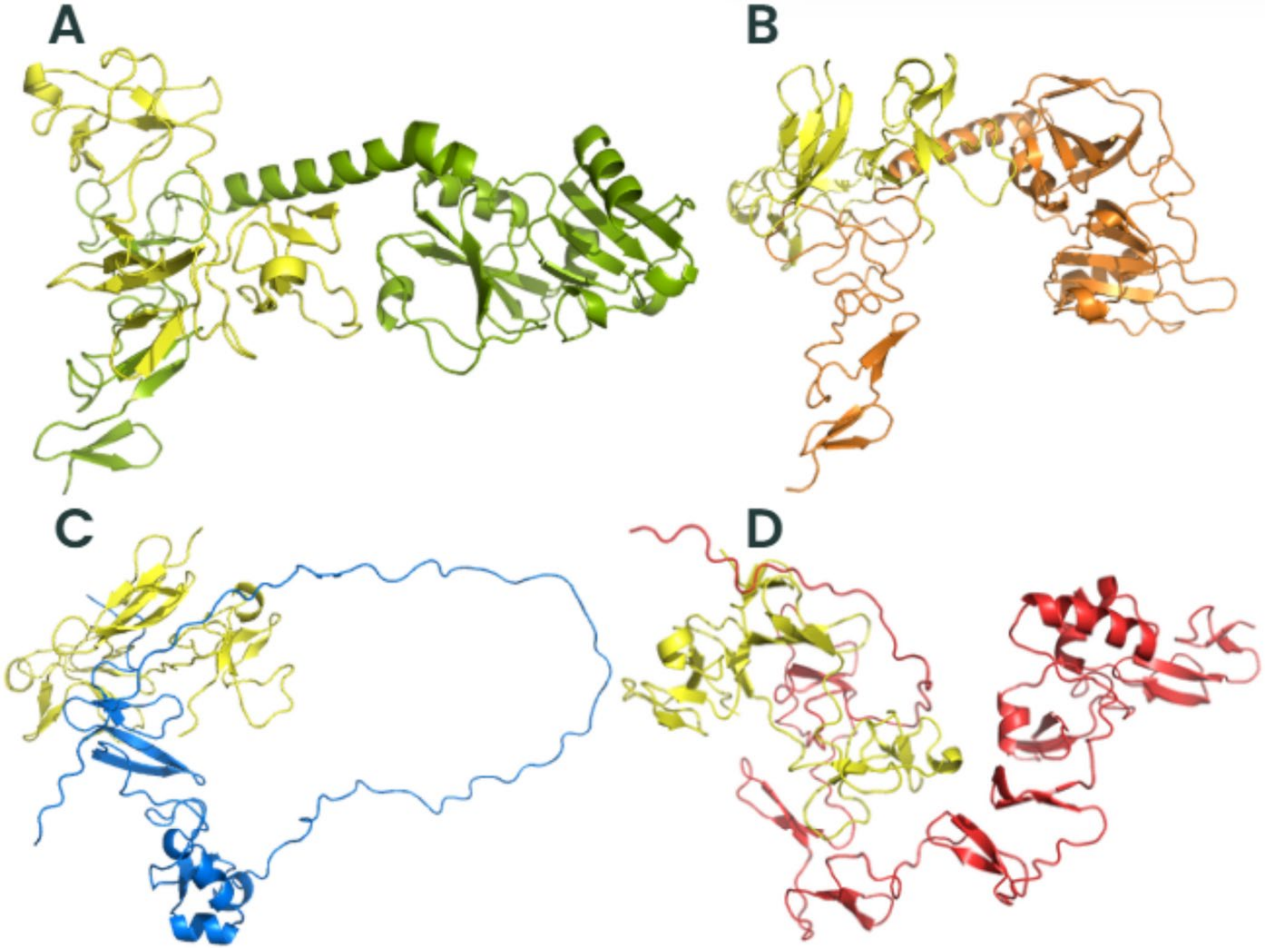
Flocculins binding to the human hemoglobin protein in brown. **A** Dha1p in green; **B** Dha2p in orange; **C** Cpl1p in blue; and **D** Cfl1p in red

**Fig. 9:**
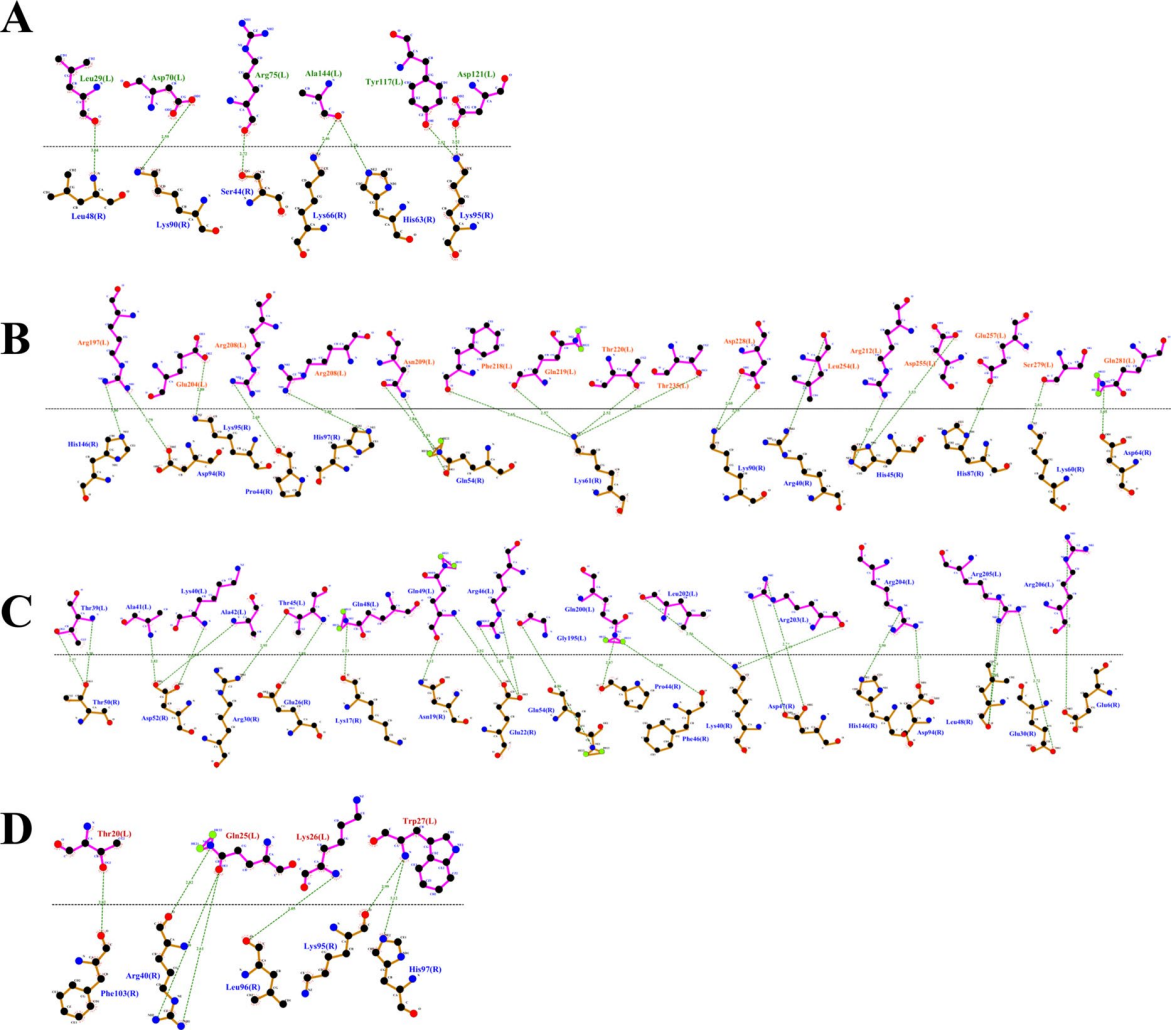
Binding between amino acid residues of human hemoglobin in blue, and those of the fungal flocculins. **A** Dha1p in green; **B** Dha2p in orange; **C** Cpl1p in blue; and **D** Cfl1p in red

For hemoglobin (Figs. 8 and 9), Dha1p bounded through N-terminal and central residues including Thr117, Asp121, and Ala144 within the PriA/Cpl1_fungi domain. Dha2p formed extensive contacts within both the PriA/Cpl1_fungi domain (Arg197, Glu204, Arg208, Asn209, Arg212, Phe218, Gln219, Thr220, Asp228) and the Cpl1-like domain (Thr235, Leu254, Asp255, Glu257, Ser279, Gln281). For Cpl1p, interactions involved N-terminal residues (Arg46, Gln48, Gln49), the PriA/Cpl1_fungi domain, and the Cpl1-like region (Gly195, Gln200, Leu202, Arg203, Arg204, Arg205, Arg206). Cfl1p exhibited interactions predominantly within its N-terminal region.

**Fig. 10:**
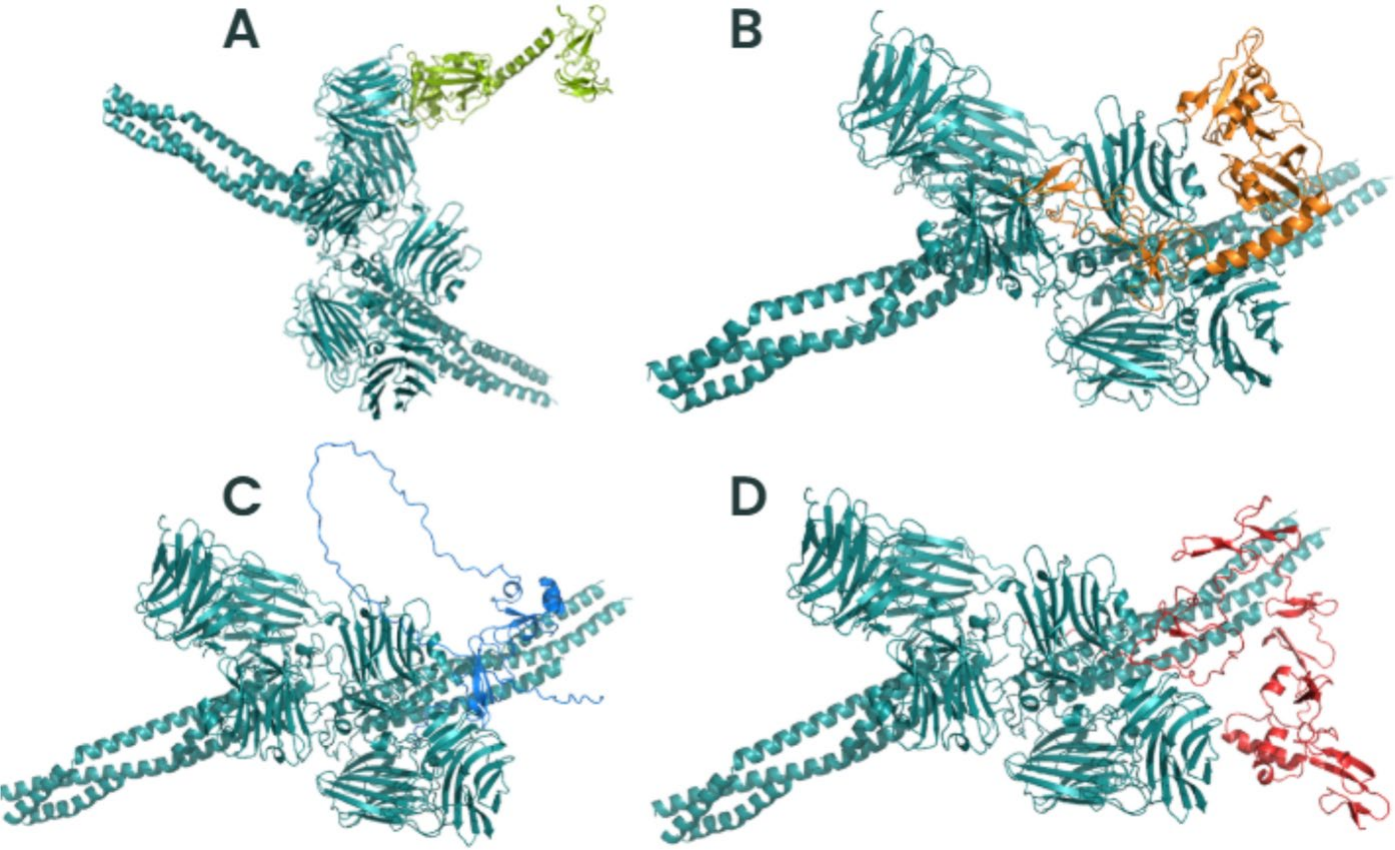
Flocculins binding to the human laminin protein in cyan-green. **A** Dha1p in green; **B** Dha2p in orange; **C** Cpl1p in blue; and **D** Cfl1p in red

**Fig. 11:**
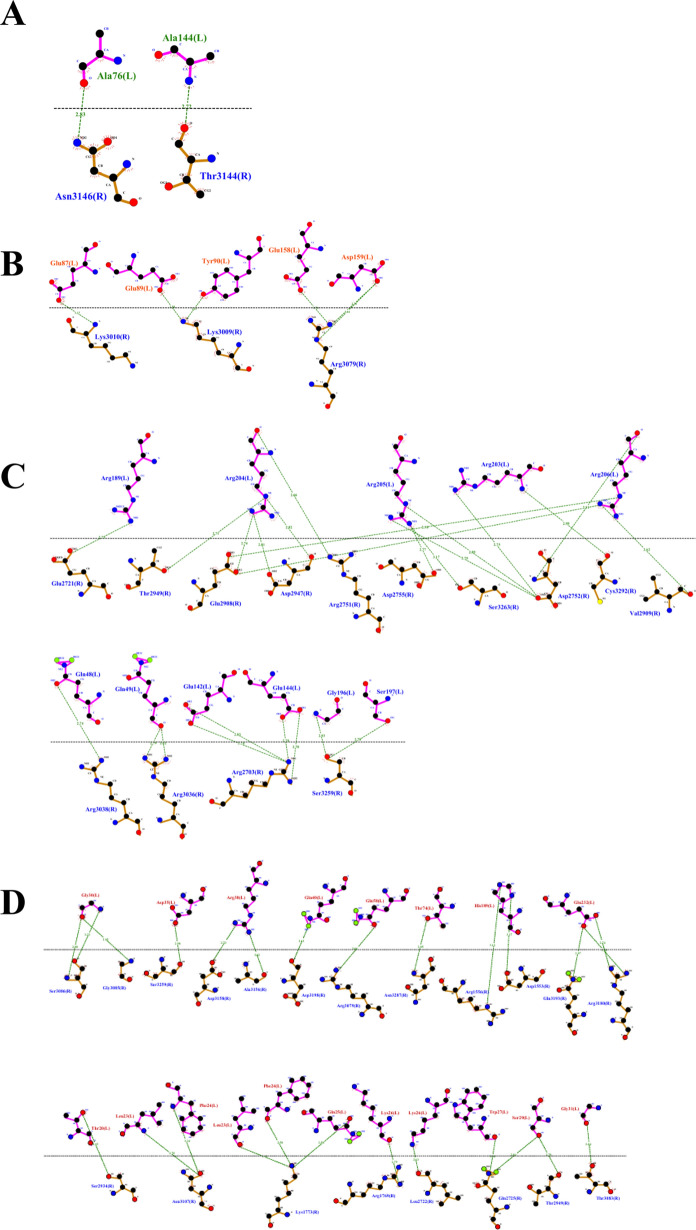
Binding between amino acid residues of human laminin, in blue, and those of the fungal flocculins. **A** Dha1p in green; **B** Dha2p in orange; **C** Cpl1p in blue; and **D** Cfl1p in red

Regarding laminin (Fig. 10 and 11), Dha1p formed limited interactions, with residues located in both the N-terminal region and the PriA/Cpl1_fungi domain (Ala144). In Dha2p, the interface involved residues in the N-terminal region and within the conserved domain (Glu158, Asp159). Cpl1p interacted via residues from both conserved domains, including Gln48 and Gln49 (PriA/Cpl1_fungi), and Glu142, Glu144, Gly196, Ser197, Arg189, Arg203, Arg204, Arg205, Arg206 (Cpl1-like). Cfl1p engaged laminin through residues predominantly located in the N-terminal region and the PriA/Cpl1_fungi domain (His189, Glu232).

### *Trichosporon asahii* CBS2479 Exhibits a Higher Proportion of Yeast Cells Relative to Hyphal Forms Present in L2585

Microscopic analysis revealed significant variability in the morphological characteristics of *T. asahii* strains. Specifically, the reference strain *T. asahii* CBS2479 displayed a predominance of yeast cells, with a yeast-to-hyphae ratio of 4:1. In comparison, both the *T. asahii* CBS7631 and the clinical strain L773 exhibited a more balanced ratio of 3:2. Conversely, the clinical strain L2585 demonstrated a strikingly different profile, with a yeast-to-hyphae ratio of 1:4 (Supplementary Fig. 2). These findings highlight the inherent morphological diversity within *T. asahii*, which may have implications for understanding strain-specific growth dynamics and pathogenicity.

### Flocculin Gene Expression are Reduced Compared to the Reference Strain CBS2479

Quantitative RT-PCR analyses revealed significant differential expression patterns of the *DHA1*, *DHA2*, *CPL1*, and *CFL1* flocculin genes across four *T*. *asahii* strains (CBS2479, CBS7631, L2585, and L773) under planktonic and biofilm conditions.

**Fig. 12:**
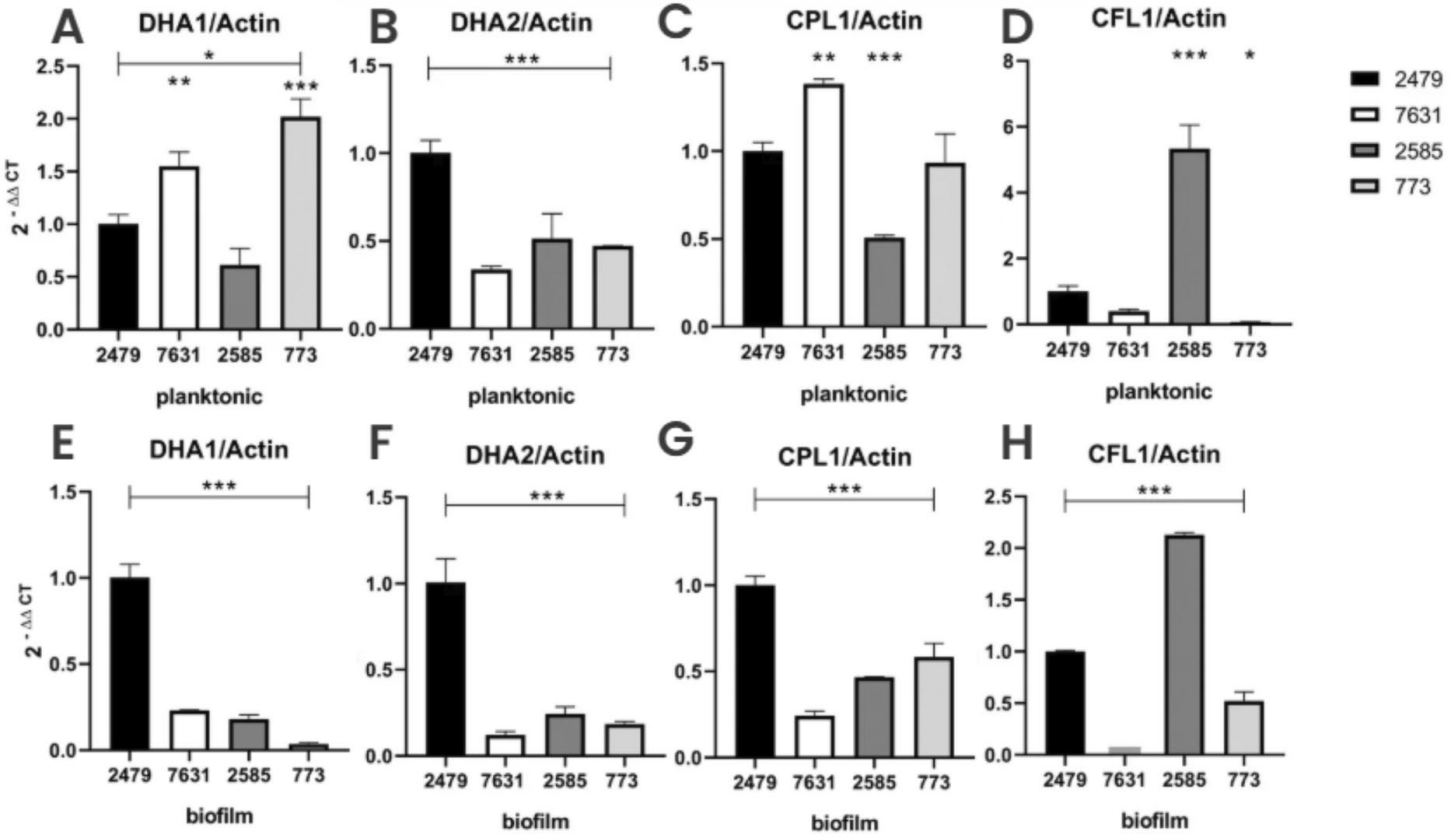
Expression of flocculin genes under planktonic and biofilm conditions, relative to the reference strain CBS2479, with significance levels indicated as *p* < 0,05 (*), *p* < 0.01 (**) e *p* < 0.001 (***). **A** Dha1p in planktonic; **B** Dha2p in planktonic; **C** Cpl1p in planktonic; **D** Cfl1p in planktonic; **E** Dha1p in biofilm; **F** Dha2p in biofilm; **G** Cpl1p in biofilm; **H** Cfl1p in biofilm

In planktonic cells, *DHA1* and *CPL1* expressions were markedly upregulated in strains CBS7631, and L773 compared to the reference strain CBS2479 (*p* < 0.001), with strain L773 exhibiting the highest transcript levels (approximately 2.2-fold increase; *p* < 0.001). On the other hand, *DHA2* expression was significantly elevated in strain CBS2479 relative to the other strains (*p* < 0.001) (Fig. 12). Considering strain L2585 under planktonic growth, the *CFL1* expression demonstrated a robust induction (nearly fourfold; *p* < 0.001). These data indicate that the transcription of these genes is selectively suppressed under conditions favoring the yeast form.

Under biofilm growth conditions, *DHA1*, *DHA2* and *CPL1* dramatically reduced their expression across all strains (*p* < 0.001) when compared to L2479. Considering *CFL1* expression, it remained highly induced in strain L2585 under biofilm conditions (*p* < 0.001) when compared to CBS2479, consistent with the planktonic profile. This suggests that *CFL1* is preferentially upregulated in this strain regardless of the growth state (Fig. 12).

### *DHA1 *and *CFL1* are Overexpressed Under Biofilm Condition

**Fig. 13:**
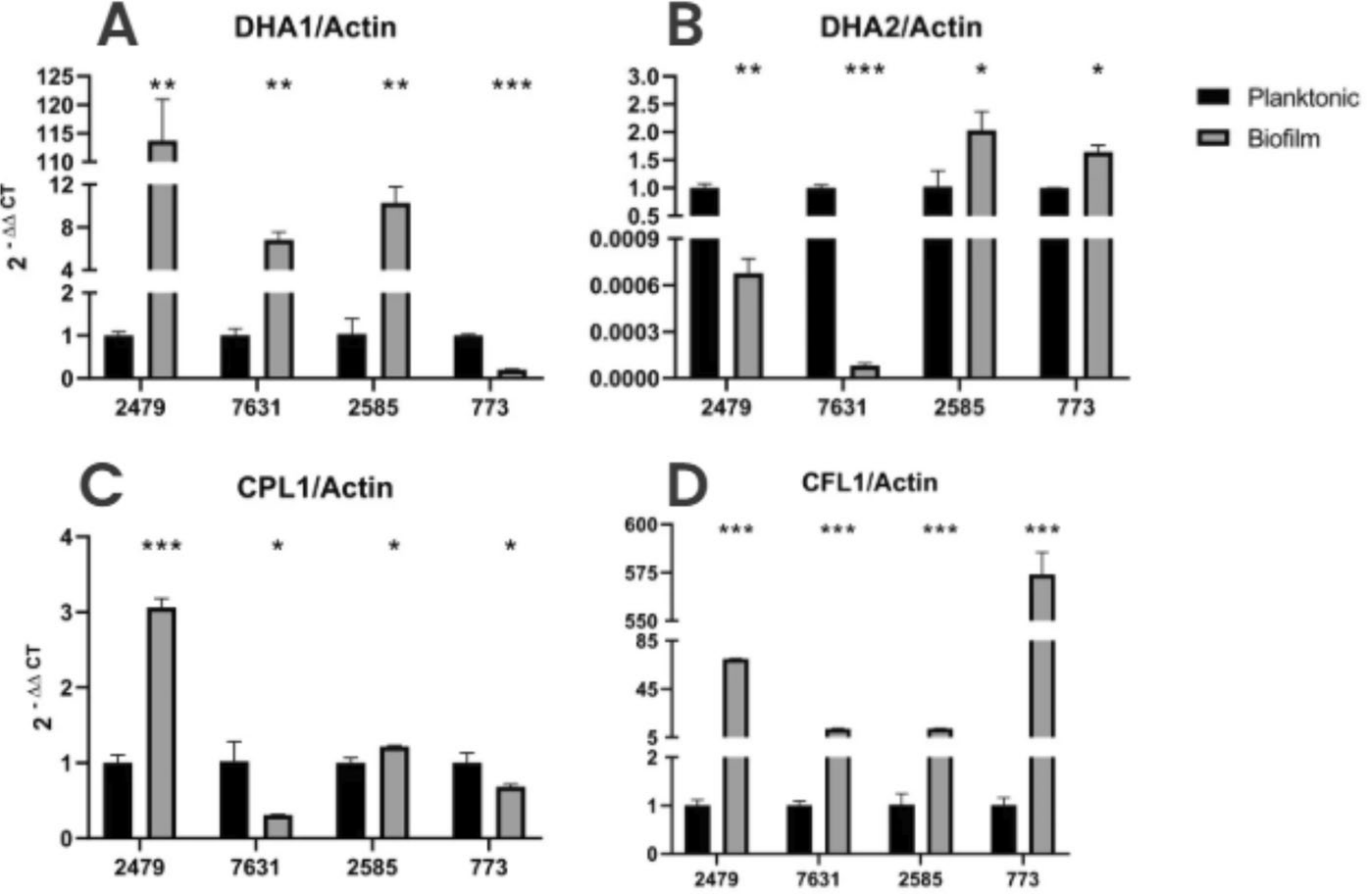
Comparison of flocculins expression under planktonic and biofilm conditions, with significance levels indicated as *p* < 0.05 (*), *p* < 0.01 (**) e *p* < 0.001 (***). **A** Dha1p; **B** Dha2p; **C** Cpl1p; **D** Cfl1p

Gene expression analysis revealed pronounced transcriptional shifts in response to biofilm growth across all four strains. *DHA1* expression was strongly induced under biofilm conditions in all strains compared to planktonic growth. The most striking increase was observed in strain L2479, which displayed an over 100-fold upregulation (*p* < 0.001). Similarly, strains 7631, 2585, and 773 exhibited significant induction, albeit to a lesser extent (*p* < 0.01 to *p* < 0.001) (Fig. 13). Similarly, *CFL1* displayed a dramatic and uniform upregulation in biofilms relative to planktonic growth across all strains (*p* < 0.001). Particularly in strain L773, *CFL1* expression increased by more than 500-fold. This persistent and robust activation of both genes under biofilm conditions suggest potential roles in biofilm formation or cell-to-cell adhesion.

The *DHA2* was suppressed (*p* < 0.001) in biofilms of both CBS strains. In contrast, clinical strains L2585 and L773 exhibited an inverse profile, with slightly upregulation in biofilms (*p* < 0.001). These findings suggest that *DHA2* is subject to distinct regulatory circuits depending on the strain, not the predominant morphology. The *CPL1* gene expression profile characterized by high expression in biofilm forming cells of *T. asahii* CBS 2479, that present more yeasts than hyphae compared to the other three strains, with a threefold increase (*p* < 0.0001). This downregulation in the biofilm condition suggests that *CPL1* is preferentially expressed during the yeast-form even during biofilm formation.

## Discussion

In this study, we identified and performed in silico characterization of four *Trichosporon asahii* flocculin-like proteins—Dha1p, Dha2p, Cpl1p, and Cfl1p—with structural features and predicted post-translational modifications consistent with adhesin function. Sequence comparisons with *Cryptococcus neoformans* flocculins revealed that they are homologous with varying degrees of identity and coverage, suggesting a conserved yet species-adapted repertoire of adhesion-related proteins among basidiomycetous yeasts [19, 22, 30, 41].

All four proteins presented glycosylation, signal peptides, or GPI modifications consistent with extracellular localization, a common characteristic of fungal adhesins already described by [1, 6, 9, 24].

Notably, Dha2p exhibited a GPI anchor and Dha1p was uniquely mannosylated, modifications that have been previously implicated in the stabilization and anchoring of cell wall proteins in fungi. The detection of Cpl1-like domains and intrinsically disordered regions in Cpl1p and Cfl1p aligns with findings in other yeast adhesins, supporting their role in flexible, multivalent host interactions [19, 41].

Conservation of adhesin domains are well known in the *ALS* family of *C. albicans* adhesins. For instance, Als1p, Als3p, and Als5 exhibit highly similar N-terminal domain profiles, characterized by conserved patterns of steric architecture, hydrophobic surface distribution, and electrostatic potential, determined by 3D modelling [15, 20, 31, 37, 38].

Molecular docking analyses revealed potential interactions between flocculins and different human proteins, which is characteristic of adhesins. It is known that Hwp1p of *Candida albicans* is a GPI-anchored, proline/glutamine-rich adhesin that becomes covalently cross-linked to mammalian epithelial surfaces via host transglutaminase, mediated through its N-terminal glutamine-rich repeat motifs [25, 28, 39]. Also, enolase, a classic enzyme of the glycolytic pathway that is present in the *C. albicans* cell wall surface with adhesin function binds in cadherin in endothelial cells, laminin in the basal membrane, fibronectin in the extracellular matrix and also plasminogen [8, 11]. Taken together, the molecular features of *T. asahii* flocculins, although based on in silico analyses, are of critical relevance, as adhesins represent promising targets for immunotherapeutic and vaccine strategies aimed at blocking fungal adhesion and preventing subsequent host colonization and infection [7, 11, 36].

Our expression analyses reveal coordinated, strain-specific regulatory programs governing *DHA1*, *DHA2*, *CPL1*, and *CFL1* expression in response to growth-state transition. Notably, *DHA1* and *CFL1* are broadly induced in the biofilm state, while *CPL1* is preferentially expressed in planktonic conditions and repressed during biofilm formation. *DHA2* retains high expression independent of lifestyle in certain strains. This morphology-dependent regulatory pattern is consistent with trends observed for other adhesin families, where gene expression is tightly coupled to specific growth forms. In *Candida albicans*, the adhesin genes *ALS3* and *ALS1* are strongly induced during hyphal development and early biofilm formation, then repressed during maturation [21, 34, 37, 43].

The consistent induction of *CFL1* under biofilm conditions across strains suggests a similar central adhesive function. Conversely, *CPL1* is enriched in yeast-phase cells and repressed in biofilms, echoing the behavior of yeast-specific adhesins in *C. albicans*. In *S. cerevisiae*, the *FLO* gene family—especially *FLO11*—regulates adhesion and biofilm/mat formation; its disruption impairs these phenotypes, again mirroring *CFL1* upregulation in biofilm contexts [12]. Thus, *CFL1* likely functions akin to *ALS3* or *FLO11* as a biofilm adhesin, while *CPL1* appears specific to the yeast form.

In *Cryptococcus neoformans*, the secretory protein family including *DHA1*, *DHA2*, and *CFL1* shows cell-type and morphotype–specific expression. *DHA1* displays strong transcript expression and intracellular punctate localization in yeast-phase cells grown on rich medium, and further becomes enriched in basidia and spores during sexual development [19]. This aligns with our observation that *T. asahii DHA1* is more expressed in planktonic growth, indicating possible conserved regulatory control. Conversely, *C. neoformans DHA2* exhibits high transcript levels during sexual development on V8 medium, yet fluorescent tagging reveals its localization predominantly in yeast cells rather than hyphae [19]. Intriguingly, *CFL1* in *C. neoformans* is hypha-specific and essential for aerial hypha production and sporulation, as demonstrated by hyphal deficiency in *cfl1Δ* mutants [19]. This phenotype closely mirrors our observation of *CFL1* induction in biofilm conditions. The fact that *CFL1* is highly induced during hyphal or biofilm conditions across both basidiomycete species underscores its potential as a conserved biofilm adhesin, placing *CFL1* as a functional hub in multicellular differentiation [19].

To further support our findings, functional studies, such as gene deletion or overexpression, as well as phenotypic adhesion assays, are required to definitively confirm the roles of these proteins in host interaction, biofilm formation, and virulence. Moreover, it remains to be determined whether these flocculins contribute to immune evasion or antifungal resistance, both of which are critical in the clinical persistence of *T. asahii* [2, 23, 32].

In conclusion, our findings expand the understanding of the adhesive mechanisms of *T. asahii* and identify novel flocculin candidates with predicted structural and functional features consistent with adhesins. These proteins may serve as promising targets for the development of antifungal agents or immunotherapeutic strategies, particularly in invasive infections where treatment options remain limited and outcomes are often poor.

## Supplementary Information

Below is the link to the electronic supplementary material.

Supplementary file1 (DOCX 2980 KB)

## Data Availability

No datasets were generated or analysed during the current study.
