# Supplementary material for: In Silico Analysis and Expression Profiling of Adhesin-Like Flocculins in Trichosporon asahii
Source: Mycopathologia. 2026 Feb 2;191(1):28. doi: 10.1007/s11046-025-01045-2 (PMC12864254; doi:10.1007/s11046-025-01045-2)
Supplement: Supplementary file 1 — Supplementary file1 (DOCX 2980 KB) [file 11046_2025_1045_MOESM1_ESM.docx]

**Supplementary data**

**Supplementary Table 1**: Primer sequences used for the amplification of *Trichosporon asahii* flocculin genes in the qPCR assay. All primers were designed with an optimal annealing temperature of 60 °C.

| Primer pair | Sequence |
| --- | --- |
| Dha1_Fw | 5'CTACCCTCCCGTCACCTCT3' |
| Dha1_Rv | 5'CCTGGGCGGCAGCAG3' |
| Dha2_Fw | 5'AGACCGCCTGCATCATTCC3' |
| Dha2_Rv | 5'GAACTCAGCATCGAGAGGAGG3' |
| Cpl1_Fw | 5'TTGAGAACGTCAAGACGGCG3' |
| Cpl1_Rv | 5'CTGCTGGGCAGACCCTC3' |
| Cfl1_Fw | 5’CAAGAAGCAGGACAACACCA3’ |
| Cfl1_Rv | 5’ATCATGCAAGCCTTGAGGTC3’ |
| Actin_Fw | 5’GGAAGGACCTCTAC GGCAAC3’ |
| Actin_Rv | 5’GCGGTGATCTCCT TCTGCAT3’ |

**Supplementary Table 2:** Ranking of protein–protein complexes formed between human serum albumin and *Trichosporon asahii* flocculins (Dha1p, Dha2p, Cpl1p, and Cfl1p), based on binding affinity scores obtained using ClusPro 2.0.

| **Flocculin** | **Cluster** | **Size** | **Center Score** | **Lowest Score** |
| --- | --- | --- | --- | --- |
| **Dha1p** | **0** | **82** | **-927** | **-956** |
| Dha1p | 1 | 57 | -911,7 | -930 |
| Dha1p | 2 | 53 | -923,2 | -964,8 |
| Dha1p | 3 | 41 | -884 | -917,6 |
| Dha2p | 0 | 93 | -891,6 | -1083,2 |
| Dha2p | 1 | 69 | -1081,1 | -1081,1 |
| Dha2p | 2 | 63 | -938,2 | -1152 |
| **Dha2p** | **3** | **61** | **-1073** | **-1207,4** |
| Cpl1p | 0 | 37 | -954,6 | -1104,8 |
| Cpl1p | 1 | 32 | -971,8 | -975,7 |
| Cpl1p | 2 | 28 | -1058,4 | -1058,4 |
| **Cpl1p** | **3** | **24** | **-1077,8** | **-1217,8** |
| **Cfl1p** | **0** | **34** | **-1265,4** | **-1431,8** |
| Cfl1p | 1 | 28 | -1268,6 | -1329,4 |
| Cfl1p | 2 | 21 | -1191,7 | -1221,2 |
| Cflp | 3 | 21 | -1187,9 | -1319,4 |

Legend: Indicated in **bold** are the best scores of the molecular docking chosen for further anlysis.

**Supplementary Table 3:** Ranking of protein–protein complexes formed between human collagen and *T. asahii* flocculins (Dha1p, Dha2p, Cpl1p, and Cfl1p), based on docking scores obtained using ClusPro 2.0.

| **Flocculin** | **Cluster** | **Size** | **Center Score** | **Lowest Score** |
| --- | --- | --- | --- | --- |
| **Dha1p** | **0** | **54** | **-957,8** | **-1211,7** |
| Dha1p | 1 | 53 | -974,9 | -1105,9 |
| Dha1p | 2 | 39 | -937,4 | -1001,5 |
| Dha1p | 3 | 38 | -986,5 | -1107,4 |
| **Dha2p** | **0** | **263** | **-1051,6** | **-1067,2** |
| Dha2p | 1 | 89 | -953,4 | -1256,9 |
| Dha2p | 2 | 85 | -993,9 | -1212 |
| Dha2p | 3 | 64 | -1007,6 | -1146,4 |
| Cpl1p | 0 | 25 | -1087,6 | -1094,4 |
| **Cpl1p** | **1** | **24** | **-1151,3** | **-1165** |
| Cpl1p | 2 | 24 | -1152,5 | -1152,5 |
| Cpl1p | 3 | 21 | -1075,8 | -1077,1 |
| Cfl1p | 0 | 23 | -1289,6 | -1378 |
| Cfl1p | 1 | 22 | -1402 | -1514,7 |
| **Cfl1p** | **2** | **22** | **-1433,3** | **-1433,3** |
| Cfl1p | 3 | 22 | -1310,7 | -1484,6 |

Legend: Indicated in **bold** are the best scores of the molecular docking chosen for further anlysis.

**Supplementary Table 4:** Ranking of protein–protein complexes formed between human fibronectin and *T. asahii* flocculins (Dha1p, Dha2p, Cpl1p, and Cfl1p), based on molecular docking scores obtained using ClusPro 2.0.

| **Flocculin** | **Cluster** | **Size** | **Center Score** | **Lowest Score** |
| --- | --- | --- | --- | --- |
| Dha1p | 0 | 89 | -855 | -968,8 |
| Dha1p | 1 | 73 | -815 | -982,4 |
| Dha1p | 2 | 66 | -851,5 | -931,8 |
| **Dha1p** | **3** | **35** | **-866,8** | **-894,7** |
| Dha2p | 0 | 132 | -963,9 | -1142,8 |
| Dha2p | 1 | 78 | -1033 | -1103,2 |
| **Dha2p** | **2** | **70** | **-1097** | **-1148** |
| Dha2p | 3 | 42 | -933,4 | -1004,1 |
| **Cpl1p** | **0** | **76** | **-942,6** | **-1217,8** |
| Cpl1p | 1 | 51 | -944,7 | -1153,4 |
| Cpl1p | 2 | 49 | -933,8 | -1074,2 |
| Cpl1p | 3 | 44 | -1039,4 | -1159,6 |
| **Cfl1p** | **0** | **60** | **-1231,4** | **-1294,5** |
| Cfl1p | 1 | 49 | -1076,9 | -1181,4 |
| Cfl1p | 2 | 43 | -1144,5 | -1252,5 |
| Cfl1p | 3 | 43 | -1093 | -1338,8 |

Legend: Indicated in **bold** are the best scores of the molecular docking chosen for further anlysis.

**Supplementary Table 5:** Ranking of protein–protein complexes formed between human hemoglobin and *T. asahii* flocculins (Dha1p, Dha2p, Cpl1p, and Cfl1p), based on molecular docking scores obtained using ClusPro 2.0.

| **Flocculin** | **Cluster** | **Size** | **Center Score** | **Lowest Score** |
| --- | --- | --- | --- | --- |
| **Dha1p** | **0** | **121** | **-963,1** | **-1004,1** |
| Dha1p | 1 | 115 | -893,6 | -1024,1 |
| Dha1p | 2 | 77 | -915,1 | -956,1 |
| Dha1p | 3 | 72 | -920,1 | -960,1 |
| Dha2p | 0 | 81 | -988,6 | -1113,7 |
| **Dha2p** | **1** | **79** | **-1149,6** | **-1154,1** |
| Dha2p | 2 | 69 | -1109,1 | -1139,6 |
| Dha2p | 3 | 53 | -1020,7 | -1108,9 |
| **Cpl1p** | **0** | **56** | **-999** | **-1089,5** |
| Cpl1p | 1 | 50 | -1078,8 | -1219,5 |
| Cpl1p | 2 | 46 | -950,4 | -1166,3 |
| Cpl1p | 3 | 44 | -1112,3 | -1112,3 |
| **Cfl1p** | **0** | **40** | **-1368,2** | **-1368,2** |
| Cfl1p | 1 | 37 | -1245,5 | -1321,4 |
| Cfl1p | 2 | 35 | -1315,8 | -1459,5 |
| Cfl1p | 3 | 33 | -1382 | -1382 |

Legend: Indicated in **bold** are the best scores of the molecular docking chosen for further anlysis.

**Supplementary Table 6:** Ranking of protein–protein complexes formed between human laminin and *T. asahii* flocculins (Dha1p, Dha2p, Cpl1p, and Cfl1p), based on molecular docking scores obtained using ClusPro 2.0.

| **Flocculin** | **Cluster** | **Size** | **Center Score** | **Lowest Score** |
| --- | --- | --- | --- | --- |
| **Dha1p** | **0** | **48** | **-1207,7** | **-1218,5** |
| Dha1p | 1 | 25 | -916,3 | -1070 |
| Dha1p | 2 | 25 | -981,1 | -1082,7 |
| Dha1p | 3 | 21 | -1000,2 | -1100,6 |
| Dha2p | 0 | 25 | -1107,8 | -1107,8 |
| **Dha2p** | **1** | **21** | **-1160,1** | **-1198** |
| Dha2p | 2 | 20 | -1158 | -1264,8 |
| Dha2p | 3 | 20 | -1029,2 | -1029,2 |
| Cpl1p | 0 | 54 | -1161,9 | -1236,7 |
| **Cpl1p** | **1** | **43** | **-1240,5** | **-1404,2** |
| Cpl1p | 2 | 33 | -1118,2 | -1165 |
| Cpl1p | 3 | 32 | -1119 | -1261 |
| Cfl1p | 0 | 21 | -1282,1 | -1446,5 |
| Cfl1p | 1 | 21 | -1466,3 | -1466,3 |
| **Cfl1p** | **2** | **20** | **-1541,5** | **-1605,4** |
| Cfl1p | 3 | 20 | -1492,7 | -1492,7 |

Legend: Indicated in **bold** are the best scores of the molecular docking chosen for further anlysis.

**Supplementary Figure 1:** Maximum Likelihood tree of DNA sequences from homologous flocullin genes from *C. neoformans* (Cn) and *T. asahii* (Ta). The alignment contained 1,396 positions analyzed. Bootstrap values calculated from 100 pseudoreplicated alignments are shown at the nodes. GenBank assession numbers identify each sequence. The bar represents 0.1 substitutions per site.


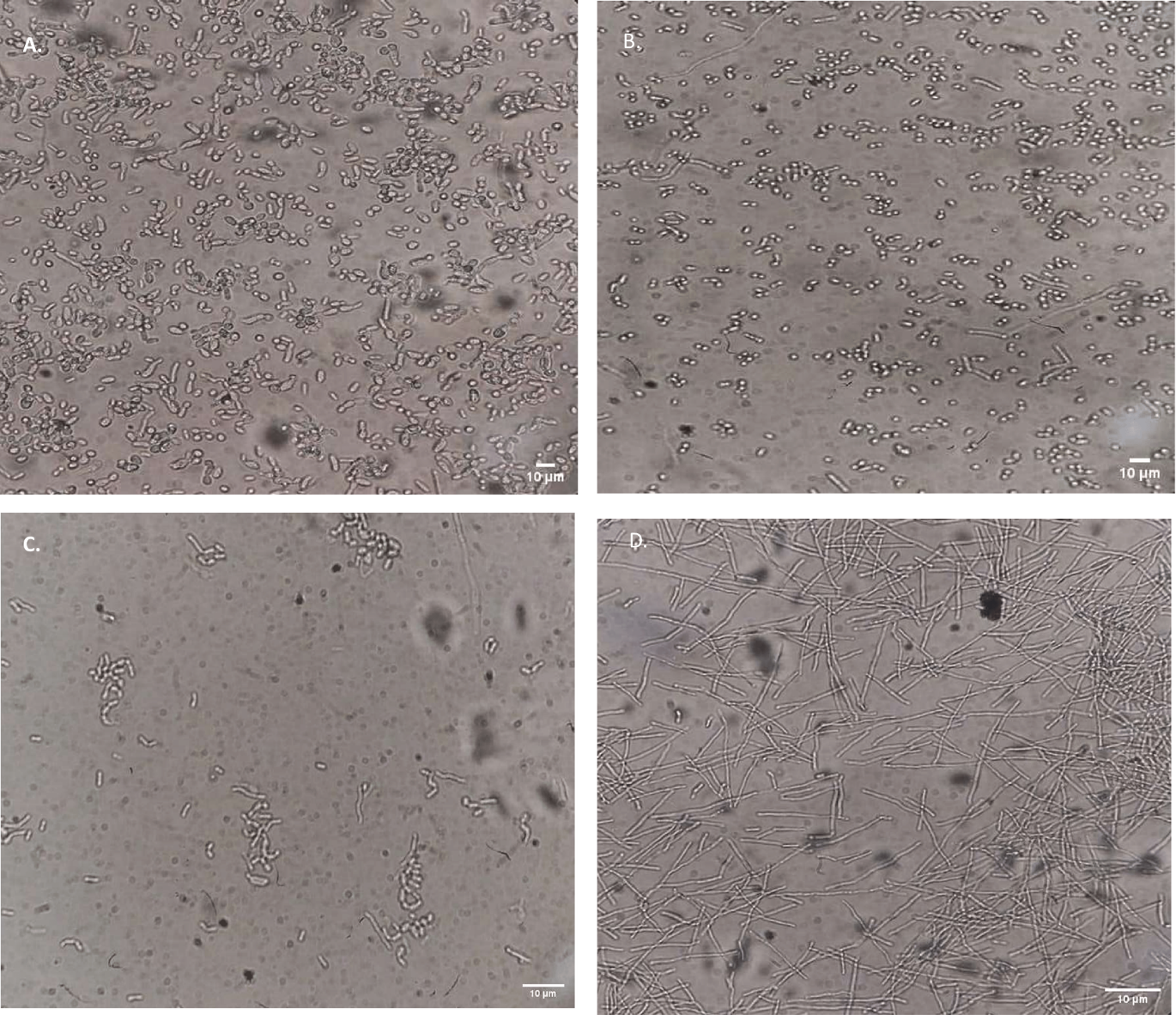


**Supplementary Figure 2:** Bright-field optical microscopy of *Trichosporon asahii* strains: (A) *T. asahii* CBS 2479, (B) *T. asahii* CBS 7631, (C) *T. asahii* L773, and (D) *T. asahii* L2585. 400 X magnification.
